# Microwave Heating Characteristics on Lipid Quality in Sterilized Rainbow Trout (*Oncorhynchus mykiss*) Using Designed Heating Processing

**DOI:** 10.3390/foods13172727

**Published:** 2024-08-28

**Authors:** Ji Zhang, Donglei Luan

**Affiliations:** 1 College of Food Science and Technology, Shanghai Ocean University, Shanghai 201306, China; kg3630@163.com; 2 Engineering Research Center of Food Thermal-Processing Technology, Shanghai Ocean University, Shanghai 201306, China

**Keywords:** traditional thermal process, lipid quality, fatty acid, thermal processing level

## Abstract

The aim of this study was to simulate microwave heating characteristics to investigate the lipid quality in rainbow trout, including the impact of the heating rate, maximum temperature, and thermal processing level on the extent of lipid oxidation and on the fatty acid extraction coefficient. Increasing F_0_ from 3 to 6 min improved fatty acid retention at high heating rates but led to a decrease in the measured results at low heating rates. Elevated thermal processing levels and maximum temperatures were observed to intensify the oxidation. At F_0_ = 3 min, an increase in maximum temperature led to an increase in the total lipid extraction coefficient but a decrease in the fatty acid extraction coefficient. However, an increase in maximum temperature resulted in a decrease in both extraction coefficients when F_0_ was 6 min. The coefficient spectra of fatty acid extraction obtained from the microwave and traditional heat treatments showed nonparallel trends, confirming the presence of non-thermal effects during microwave thermal processing. In conclusion, compared to conventional heat treatment methods, microwave processing has significant potential for enhancing the lipid quality of ready-to-eat rainbow trout products and effectively reducing production costs.

## 1. Introduction

High-temperature short-term processing offers minimal heating times to retain the nutritional value of processed foods as much as possible and has the potential to ensure microbial safety [1]. In traditional thermal processing that uses hot water or steam as the heating medium, the heat transfer rate is limited by the low heat conductivity of solid food products. Therefore, novel processing technologies must be developed to increase the heat conductivity rate during thermal processing. Microwaves can provide volumetric heating to food products via alternating electric fields, providing rapid heating rates and significantly improving product quality in a short time [2,3].

Microwave heating can make a sample reach its target temperature in a very short time, greatly reducing the heating time. Unlike heat conduction and heat convection, the overall heating mode has less impact on the product quality and interior structure [4]. Microwaves can also enhance the perception of saltiness, which plays an important role in research and development in respect of low-salt production [2]. However, for low-moisture foods, such as raisins and Ganoderma spores, more research and pre-experiments are needed to improve the thermal efficiency of sterilization.

Microwave processing is said to induce a non-thermal effect that can effectively eliminate microorganisms while preserving the nutritional content of the target food [5,6,7]. The implementation of a novel verification approach, referred to as the double-side approximation method (DSAM), effectively mitigates thermal interference in microwave processing, thereby allowing exploration of the non-thermal effects of microwaves on nutrients [6]. The DSAM cannot generate compelling data if fluctuations in nutrient levels exhibit chaotic patterns under high-temperature conditions.

In addition to the characteristic rapid heating rate, during the microwave thermal process, the surface of the sample exhibits nonuniform heating, which leads to various thermal processing levels across different regions. The corresponding localization is typically determined using a chemical coding method, which utilizes model foods with identical dielectric properties [8].

The rapid heating rate of microwaves leads to an increase in the maximum temperature. When exploring the non-thermal effects of microwave thermal processing in the food industry through the DSAM, uncertainty often surrounds the desired maximum temperature. Without understanding how fluctuations in the maximum temperature affect lipid quality, it is difficult to confidently determine whether changes in lipid quality can be attributed to varying maximum temperatures or other factors. Therefore, it is crucial to examine the influence of different maximum temperatures.

Rainbow trout, with its abundant nutrients and delicate flavor, is widely popular on a global scale [9]. Due to its robust adaptability and rapid growth rate, rainbow trout has become the preferred species for numerous aquaculture projects. Although a small proportion of rainbow trout is consumed raw, the majority is subsequently processed and packaged in canned form [10]. During food processing, retort sterilization is the most prevalent method of sterilization [3].

However, retort sterilization leads to a substantial and rapid decrease in the nutritional quality of rainbow trout, particularly with regard to unsaturated fatty acids. Unsaturated fatty acids exhibit pronounced susceptibility to high temperatures and are prone to hydrolysis or oxidation, generating aldehydes, esters, alcohols, and other related oxidation products [11]. To improve the nutritional value for consumers, manufacturers often incorporate additional nutrients, such as olive oil or sunflower oil, which contain natural antioxidants, into cans prior to sterilization [12,13]. These methods not only increase the content of beneficial ingredients but also reduce internal lipid oxidation, thus improving the overall canned food production quality [14]. However, these supplementary nutrients can increase the cost of canned foods due to factors such as raw material procurement and formulation adjustments. Consequently, if the primary nutrients of canned products are retained during thermal processing, substantial costs can be reduced making the product more appealing.

The objective of this study was to investigate the impact of varying heating rates, maximum temperatures, and thermal processing levels on lipid quality parameters, such as the peroxide value (POV), reactive thiobarbituric acid reactive substances (TBARs), the acid value (AV), and the efficiency of fatty acid extraction. The fatty acid profile was analyzed utilizing gas chromatography–mass spectrometry (GC–MS). Additionally, a comparative analysis between microwave treatment and traditional heat treatment was performed to confirm the potential influence of the non-thermal effect on lipid quality.

## 2. Materials and Methods

### 2.1. Sample Preparation

**Figure 1:**
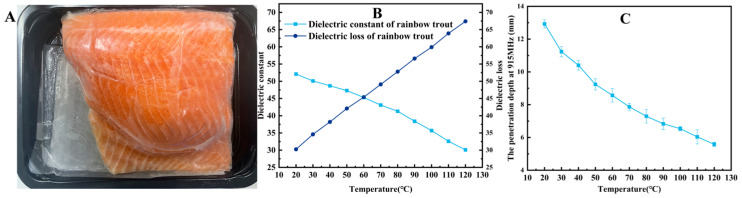
Basic data in respect of rainbow trout. (**A**) The abdominal position of the rainbow trout samples used in the experiment. (**B**) The dielectric constants of rainbow trout at 915MHz. (**C**) The dielectric losses of rainbow trout at 915 MHz.

Frozen rainbow trout was purchased from a reputable store located in Ningbo, Zhejiang province, and stored at a temperature of −18 °C (Figure 1A). The sample was placed at a temperature of 4 °C for 10 h to ensure complete thawing. The central area of the abdomen of the rainbow trout, with dimensions of 60 × 40 × 6 mm, was extracted for the following experiments. Considering the rapid heating characteristics of microwaves, the thickness of the flesh in the microwave-processing group was adjusted to 16 mm. A temperature sensor was used to monitor the time–temperature and time profile of the flesh through the tip of the sensor. In the experiment, a sensor (PICO VACQ, TMI-ORION, Castelnau le Lez, France) was inserted into a specific cold spot of the flesh, which was then enclosed along with the sensor in a retortable pouch (Yiben, Shijiazhuang, China) through vacuum sealing (Henkelman Inc., CK’s-Hertogenbosch, The Netherlands) [15]. The results of all the thermal processing parameters were based on the recording and analysis of subtle temperature fluctuations in the cold spot.

### 2.2. Traditional Thermal Processing System

The traditional system comprises two components: an oil bath system equipped with adjustable temperature control and a hermetically sealed aluminum alloy vessel designed to withstand high pressures. Dimethyl silicone oil serves as a heating medium to rapidly increase the temperature of water within a container by heating its outer surface, thus achieving the objective of heating the target food [8]. In our system, the container was hermetically sealed with six screws, allowing the internal waterbed temperature to reach up to 132 °C. In addition, a thermometer was integrated into the closed system for the real-time monitoring of the internal water temperature. After the completion of the thermal treatment, the sample was rapidly quenched in ice water to cool it. The container was then opened, and the sensor was extracted to read the recorded time–temperature profile. Specific parameters for the design of the thermal process are listed in Table 1.

#### 2.2.1. Different Oil Temperatures

In this experiment, distinct heating rates were achieved by employing dimethyl silicone oil at different temperatures: 128 °C, 148 °C, 168 °C, and 188 °C. The hermetically sealed container was immersed in an oil bath at various temperatures to achieve various heating rates that were subsequently utilized to explore the effects of the heating rate on the quality of the lipids.

#### 2.2.2. Different Maximum Temperatures

Waterbed temperature parameters of 122 °C and 132 °C were used. Thermal energy was transferred from the high-temperature waterbed to the packed flesh. The effect of temperature on the FA extraction coefficient of rainbow trout filets was determined through comparison.

#### 2.2.3. Different Thermal Processing Levels

The thermal processing level directly determines the stability duration of ready-to-eat products. In commercial production, F_0_ can be selected as either 3 or 6 min. This calculation can be performed using Equation (1).

(1) $$F_{0}=\int_{0}^{t}10^{\frac{T\left(t\right)-121.1}{z}}dt$$

T(t) represents the time–temperature curve (°C) of the cold-spot position during thermal processing, where t denotes the processing time in minutes and z represents the z-value of Botulinum type A and Botulinum type B (hydrolyzed protein) spores processed in low-acid foods; the average temperature typically ranges around 10 °C [16].

An interaction experiment with different F_0_ values and heating rates was designed to explore the influence of the thermal processing level on lipid quality under different heating rates.

### 2.3. Microwave-Assisted Thermal Sterilization

The single-model microwave-assisted sterilization system was independently developed by Shanghai Ocean University (Shanghai, China) and comprised loading, microwave, holding, and cooling chambers [6]. The temperature of the circulating water in the microwave and holding chambers was set at 122 °C with a fixed power of 5000 W from the 915 MHz magnetron (SANLE CK-611, Nanjing, China). The packed samples were placed in a loading box and transferred to a loading chamber for continuous microwave sterilization.

### 2.4. Dielectric Properties of Rainbow Trout at 915 MHz

The dielectric constant and dielectric loss of flesh were measured using an open-ended probe (Agilent N1501A, Agilent Technologies Inc., Santa Clara, CA, USA) in a network analyzer (Agilent E5071C, Agilent Technologies Inc., Santa Clara, CA, USA). The flesh was placed into a temperature-controlled holder that was connected to the oil bath treatment system and the probe was inserted and securely positioned. Once the oil bath reached the specified temperature, the flesh sample was assessed using a probe to determine the dielectric properties of rainbow trout. The frequency was 915 MHz and the temperature range spanned from 20 to 120 °C. The dielectric properties were continuously measured at 10 °C intervals [17].

### 2.5. Lipid Extraction

First, the heat-treated flesh and its liquid were transferred from the retortable pouch into a 250 mL beaker for immediate homogenization after thermal processing to obtain a homogeneous mixture while simultaneously homogenizing the raw sample. A 9.0 g mixture was placed in a 250 mL conical flask with a cover and mixed with a chloroform–methanol mixture (2:1, *v*/*v*) at a ratio of 1:20. After homogenization, the mixture was left to stand at 4 °C for 24 h and then filtered. The filtrate was mixed with 30 mL of 0.9% NaCl solution and shaken for 3 min to promote layer separation. The mixture was then transferred to a separate funnel and allowed to stand for 3 h. The bottom chloroform layer was collected and dried over anhydrous sodium sulfate. Finally, the total lipid residue was obtained via rotary evaporation at 40 °C for 10 min [18].

### 2.6. Determination of Lipid Oxidation

The Soxhlet extraction method was used to extract crude fat from the rainbow trout. The peroxide value was determined according to the method proposed by Wang et al. [19], whereas the measurement of thiobarbituric acid reactive substances (TBARs) followed the protocol developed by Jiang et al. [20]. The numerical values of TBARs are expressed in mg MDA/kg. Agnieszka’s method was used to determine the acid values [21].

### 2.7. Methylation of Fatty Acids

Following the method of ISO 12966-2: 2017 with appropriate modifications [22], 0.10 g of lipid extract was placed in a flat-bottomed flask and mixed with 6 mL of a 0.5 mol/L sodium hydroxide–methanol solution of 0.5 mol/L and 100 µL of a 10 mg/mL standard solution of non-adecanoic acid. The flask was then inserted into a reflux condenser setup and subjected to water bath treatment at a controlled temperature of 100 °C for 15 min until all internal oil droplets had completely disappeared. Subsequently, 5 mL of a 15% boron trifluoride–methanol solution was added, and refluxing was continued for an additional 5 min. Finally, chromatography-grade n-hexane (2 mL) was added, and the mixture was shaken for 2 min. After removing the flask from the water bath, the solution was supplemented with a saturated sodium chloride solution (10 mL) and transferred to a centrifuge tube for freezing centrifugation lasting 5 min to allow layer separation. The supernatant from the centrifuge tube was filtered through an organic phase filter membrane with a pore size of 0.20 µm before being transferred to a gas-phase injection bottle.

### 2.8. Analysis of Fatty Acids

Fatty acid methyl esters (FAMEs) were analyzed using a GC-MS (TRACE GC Ultra, Thermo Fisher Inc., Waltham, MA, USA) equipped with an Agilent SP-2560 capillary column (Agilent Technologies Inc., Santa Clara, CA, USA) measuring 100 m in length and 0.25 mm in internal diameter and coated with a film thickness of 0.2 μm. A mass-selective detector was used to detect analytes. Detection was performed using a flame ionization detector (Thermo Fisher Scientific, Waltham, MA, USA). The detector was used at a temperature of 260 °C, while the injector was kept at 250 °C. Fatty acid detection in GC–MS required an injection of only 1 μL into the system, with a nitrogen carrier gas flowing at a rate of 1 mL/min, and a split ratio of 45:1 was implemented. The temperature protocol used for the analysis was derived from the methodology established by Ding [23].

Because the weight inside the retortable pouch remained constant after thermal processing, the novel method exhibited significant enhancements in the extraction rates of fish oils and fatty acids, with the extraction coefficient expressed using Equation (2):

(2) $$\mathrm{Extraction}\;\mathrm{coefficient}\;(\%)=\frac{Contents\;of\;total\;lipid\;or\;fatty\;acids\;present\;in\;the\;processed\;meat\;and\;gravy}{Contents\;of\;total\;lipid\;or\;fatty\;acids\;in\;raw\;sample}$$

The results of our calculations indicate that, in this experiment, the extraction coefficient of fatty acids exceeded 100%, suggesting that heat treatment induced thermal damage to both fish tissue and lipids. Results less than 100% indicate that the fatty acids had been oxidized during thermal processing [23].

### 2.9. Statistical Analysis

The data in respect of oxidation values and extraction coefficients are expressed as means ± SD. A post hoc test and normality test were performed using SPSS v.22 software (SPSS Inc., Chicago, IL, USA). The Pearson correlation coefficients between the main MUFA and PUFA fatty acids and POV, TBAR, and AV values were used to present the correlations between the oxidation values and the extraction coefficients. All data in respect of the samples from different heat treatments were analyzed using ANOVA. The letters in the table indicate statistically significant differences (*p* < 0.05) between the raw and processed groups.

## 3. Results and Discussion

### 3.1. Dielectric Properties of Rainbow Trout at 915 MHz

The composition within the processed flesh of rainbow trout underwent alteration, leading to modified dielectric properties. The dielectric constant of the flesh of rainbow trout exhibited a decreasing trend with increasing temperature at 915 MHz (Figure 1B). Conversely, the dielectric loss demonstrated an upward trend with temperature, consistent with Hu’s previous findings [24]. The depth penetration of rainbow trout flesh gradually decreased with elevated temperature (Figure 1C). At 20 °C, the dielectric constant (ε′), dielectric loss (ε″), and microwave penetration depth of flesh were 52.0083, 30.2576, and 1.298 cm, respectively.

### 3.2. Time–Temperature Profile and F_0_

**Figure 2:**
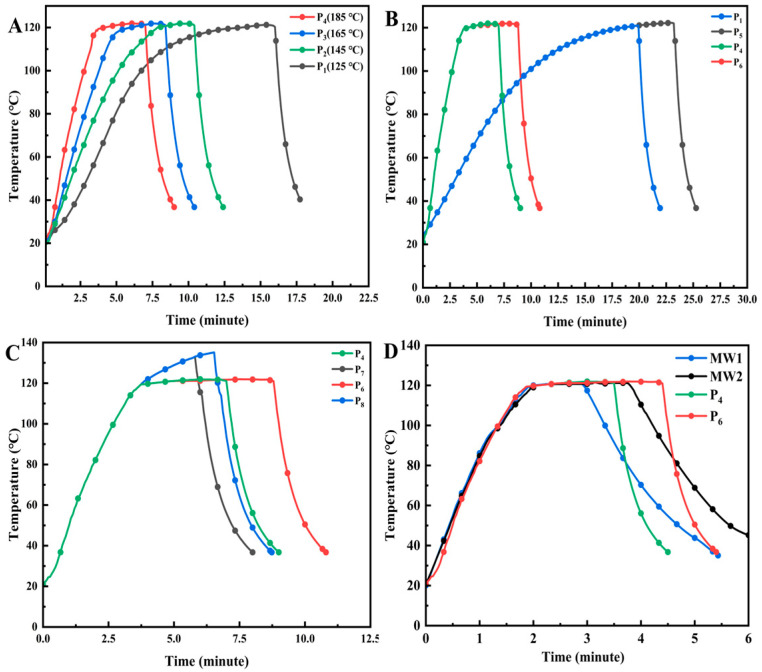
The time–temperature profiles of different heat treatments. (**A**) Different heating rates. (**B**) Different thermal processing levels. (**C**) Different maximum temperatures. (**D**) Different thermal processing methods.

The time–temperature profiles under different heat treatment parameters are shown in Figure 2. Figure 2A and Figure 2C indicate the different heating rates and maximum temperatures in the thermal process, respectively. Comparisons of the different F_0_ values are also clearly shown in Figure 2B. The heating rate of the traditional thermal processing method is difficult to match with the microwave heating rate. However, MW1 and MW2 align well with P4 and P6 in Figure 2D, while both reach an identical maximum temperature. In conventional heat treatment, where the thickness of the flesh is decreased, this profile can be utilized to simulate the trend in the time–temperature variation in microwave processing. Rapid heating can effectively mitigate the extent of structural damage to the target product and significantly enhance the efficacy of microbial inactivation [25]. Likewise, rapid heating significantly reduces the food processing time, thereby enhancing product yield and reducing costs.

**Figure 3:**
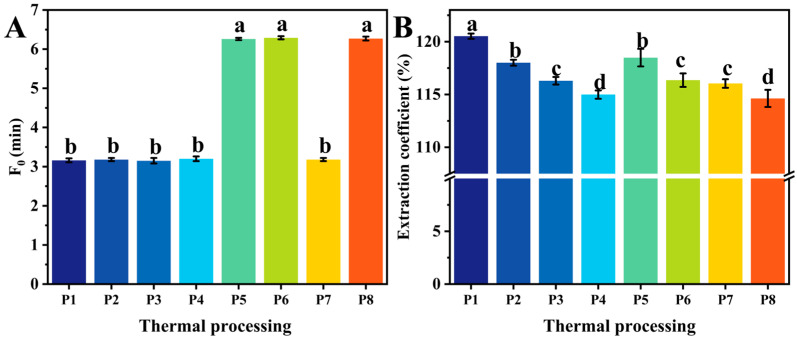
The F-values and extraction coefficients of total lipids with different heat treatment parameters. (**A**) The thermal processing levels of different traditional treatments. (**B**) The extraction coefficients of different traditional treatments. Letters a–d indicate a significant difference (*p* < 0.05) between different heating treatments.

The results in Figure 3A show the thermal processing levels under different parameters. Under different heating rates and maximum temperature conditions, the F_0_ values of the traditional thermal processing group were similar and did not differ significantly, which confirms the precision of the experimental design. When F_0_ exceeded 3 min, it could be inferred that there would be no microbial risk of low-acid food storage at room temperature [3].

### 3.3. Extraction Coefficients of Total Lipids

With an increasing heating rate (P1, P2, P3, and P4), the measured coefficients of total lipid extraction gradually decreased, as shown in Figure 3B, and the extraction coefficients decreased from 120.52% to 114.99%. With prolonged heating, the cellular architecture of the fish gradually deteriorated, leading to an increase in the measured extractable lipid content.

At low heating rates, as the thermal processing level increased (from P1 to P5), the measured total lipid extraction coefficient decreased by 1.03% (from 120.52% to 118.49%). Prolonged heating led to the oxidation of total lipids and the formation of secondary oxidation products. In contrast, at an accelerated heating rate (P4 and P6), the coefficient of total lipid extraction gradually increased from 114.99% to 116.36%.

Furthermore, increasing the water bath temperature (P4–P8) gradually reduced the coefficient of the lipid extraction coefficient, as high temperatures promote fat oxidation and decomposition, resulting in the formation of toxic substances [26,27]. Therefore, it is crucial to control the maximum temperature during thermal processing, particularly for high-lipid ready-to-eat foods.

### 3.4. Lipid Oxidation

**Figure 4:**
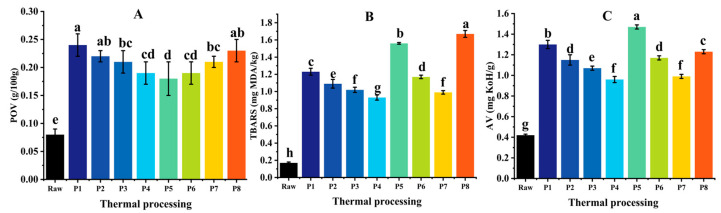
Lipid oxidation indexes of flesh obtained through different heat treatments. (**A**) The POV contents of different traditional thermal processes. (**B**) The TBAR contents of different traditional thermal processes. (**C**) The AV contents of different traditional thermal processes. Different lower letters indicate a significant different (*p* < 0.05).

The results in Figure 4A–C clearly demonstrate that an increase in the heating rate (P1, P2, P3, and P4) led to a significant reduction in the peroxide, thiobarbituric acid, and acid values. This outcome can be attributed to the increased heating rate, which resulted in a reduced thermal processing time and subsequently mitigated the level of lipid oxidation.

The POV remained unchanged with an increase in the thermal processing level (P1 and P5, P4, and P6), whereas TBARs exhibited a significant increase. A higher level of thermal processing signified an extended heating time and accelerated the transformation of the metabolite mass from primary to secondary, resulting in a reduction in the peroxide value and an elevation in the TBAR values. The heating time of P5 exceeded that of P8, yet its TBAR value was lower than that of P8. This is because the secondary oxidation products in P5 reacted with proteins and phospholipids, leading to a decrease in their contents [28].

The POVs and TBARs exhibited a substantial augmentation as the waterbed temperature rose from 122 °C to 132 °C (P4 and P7, P6, and P8), indicating that surpassing the maximum temperature limit exacerbates lipid oxidation. Concurrently, there was an upward trend in acid value (from 1.17 mg KoH/g to 1.23 mg KoH/g), suggesting that rancidity occurred in rainbow trout oil and its quality deteriorated [29].

In traditional heating methods, the outer surface of food is typically heated prior to the geometric center, resulting in elevated temperatures on the outer surface towards the end of heating, consequently accelerating the oxidation rates. In contrast, microwave heating heats the entire food sample, thereby having a lower impact on lipid quality [30].

### 3.5. Extraction Coefficients of Fatty Acids

#### 3.5.1. Different Oil Temperatures

**Figure 5:**
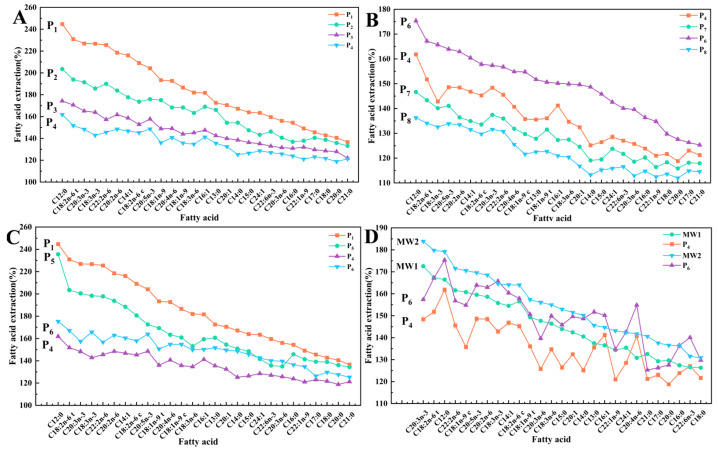
Extraction coefficients of fatty acids in rainbow trout following thermal processing using various methods and parameters. (**A**) Different heating rates. (**B**) Different thermal processing levels. (**C**) Different maximum waterbed temperatures. (**D**) Different thermal processing methods.

As shown in Figure 5A, an increase in the heating rate (P1, P2, P3, and P4) resulted in a gradual reduction in the extraction coefficients of the fatty acids. The total extraction coefficient dropped from 176.43% (P1) to 133.45% (P4). Compared to MUFAs and SFAs, the extraction coefficient of PUFAs decreased the most (from 189.75% to 141.34%) because PUFAs are sensitive to high temperatures.

The faster heating rate helps reduce thermal damage to fish cells, thereby maintaining the integrity of the cells and ultimately improving the overall quality of the product. Further, the acceleration of the heating rate significantly decreases the thermal processing time, thereby substantially enhancing the production efficiency.

#### 3.5.2. Different Thermal Processing Levels at a Low Heating Rate

As the thermal processing level increased (P1 and P5), the extraction coefficient of the fatty acids gradually decreased in the low-rate treatment group (Figure 5B). The prolonged high-temperature process resulted in the oxidation of lipids to aldehydes and ketones. At the beginning of the treatment, the lipids were extracted and oxidized concurrently. However, the extraction coefficient reached its peak value and declined irrevocably at a certain time. Higher thermal processing levels were unfavorable for the retention of fatty acids, whereas prolonged heating facilitated the oxidative degradation of fatty acids and the subsequent formation of lipid oxidation products. Luan et al. reported that a higher heat-treatment level causes a higher thermal degradation of amino acids in sterilized rainbow trout flesh, while the essential amino acid index (EAAI) is reduced simultaneously [6]. Related studies have also reported that an increase in the heating temperature and extension of the heating time causes intense lipid oxidation [31,32].

#### 3.5.3. Differences in Thermal Processing Levels at High Heating Rates

The extraction coefficients of fatty acids in the high heating rate treatment groups (from P4 to P6) gradually increased (Figure 5B). The fatty acid content gradually increased with an increase in F_0_. Because of the short heating time of P4, the flesh of the rainbow trout experienced minimal heat-induced damage, resulting in a substantial intracellular accumulation of lipids. With the prolonged heating time (P6), lipids were released in abundance, leading to an increase in the extraction coefficient. Meanwhile, the bound lipids that were difficult to extract were also gradually extracted, leading to an increase in the extraction coefficient values.

#### 3.5.4. Different Maximum Temperatures

At a certain F value, an increase in the maximum temperature resulted in a decrease in the extraction coefficients (P4, P7, P6, and P8), as shown in Figure 5C. The increase in the maximum temperature under the condition of F_0_ = 3 min resulted in a decrease in the extraction coefficient from 133.45% (P4) to 124.27% (P7). The same result was observed for F_0_ = 6 min (from 146.37% to 122.62%). Moreover, the content of all fatty acids exhibited a declining trend according to the measured data.

This phenomenon can be attributed to the elevated waterbed temperature, which subsequently increased the maximum temperature of the flesh and accelerated total lipid oxidation, ultimately leading to an increased rate of fatty acid oxidation. Therefore, it is imperative to perform preliminary experiments to determine the optimal parameters rather than blindly increasing the processing temperature to achieve the calculated F value during the thermal processing design [33,34,35].

### 3.6. Correlation Analysis of Fatty Acid Extraction Coefficients and Oxidation Values

**Figure 6:**
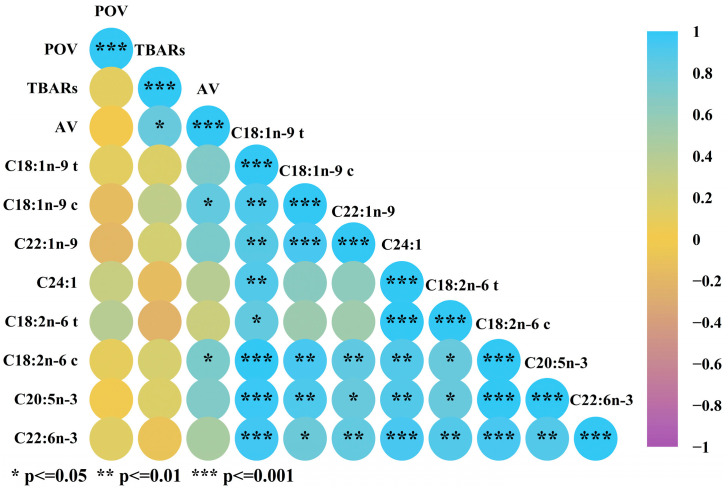
Correlation analysis of oxidation values and main fatty acid extraction coefficients in traditional thermal process group.

With the continuation of thermal processing, the lipids that could be extracted and the lipid oxidation value gradually increased. Pearson correlation analysis was used to analyze the lipid oxidation values and extraction coefficients of the main MUFAs and PUFAs under traditional heat treatment, as shown in Figure 6. The analysis revealed no significant correlation between the extraction coefficients and oxidation values. As the extent of oxidation increased, the substantial depletion of thermosensitive fatty acids led to a decrease in the extraction coefficient. However, a high correlation between the acid value and the extraction coefficient was observed, surpassing the TBAR values and POVs. On one hand, the acid value generally represents the content of free fatty acids and is positively correlated with the extraction coefficient. On the other hand, the rise in acid value means the aggravation of oxidation, resulting in rancidity [11].

Generally, the extraction coefficients of PUFAs and MUFAs exhibited consistent variations in response to changes in the thermal processing parameters. However, the specific correlation between PUFAs and MUFAs revealed inconsistency. Potential alterations in the relative proportions of fatty acids across different oxidation stages were observed [24]. The negative correlation between DHA (C22:6n-3) and TRARs indicated that thermal processing led to the degradation of DHA and a subsequent decrease in its content.

### 3.7. Microwave Thermal Processing

**Figure 7:**
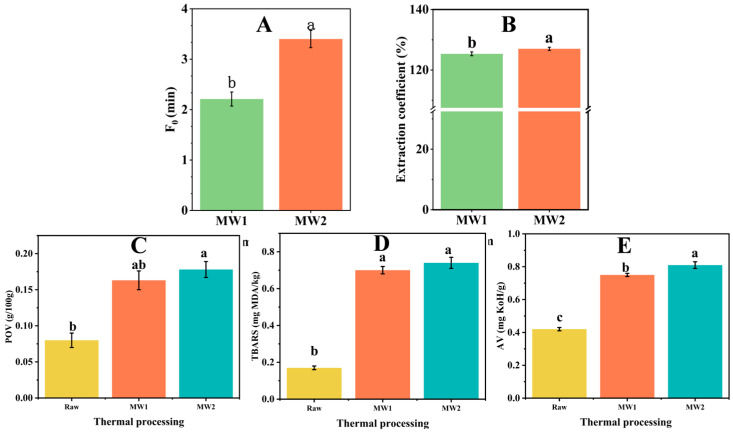
Basic data of different microwave processing groups. (**A**) Thermal processing level. (**B**) Extraction coefficients of total lipids. (**C**) POVs. (**D**) TBAR values. (**E**) AV values. Different lowercase letters in the same group indicate a significant difference (*p* < 0.05).

The F_0_ value of the MW2 group surpassed 3 min with an increase in microwave processing time and holding time (Figure 7A), meeting the requirements of a shelf-stable ready-to-eat food [3]. At approximately the same heat rate, the total lipid extraction coefficients of MW1 and MW2 were higher than those of P4. Microwave treatment efficiently separates bound lipids, resulting in a higher extraction coefficient for total lipids than traditional heat treatment [36]. Microwaves can disrupt the tissue structure of fish, making it easier for extractants to access internal lipids.

The MW-processed group demonstrated significantly lower levels of oxidative indicators (Figure 7C–E) compared to the traditional heat-treated group. The POV (0.17mg/g) and TBAR (0.70 mg MDA/kg) value of MW1 were lower than those under traditional treatments (from P1 to P8). Compared to the water bath treatment, microwaves have less impact on the quality of the target food [17]. Furthermore, an increase in the holding time (from MW1 to MW2) resulted in an increase in the thermal processing level; specifically, MW2 displayed a larger lipid value than MW1. This finding is consistent with the regular traditional thermal process (from P1 to P5). Wang et al. reported that the oxidation value of a Pacific Saury product after microwave pasteurization was lower than that under traditional retort treatment and still met the limited standard of ready-to-eat food after 7 days of storage at 37 °C [19].

Conventional thermal processing methods typically involve slow heating rates, which necessitate an increase in the target maximum temperature to achieve the desired F value. However, this approach may severely compromise the quality of the product. In contrast, microwave treatment offers a more efficient alternative by enabling the rapid attainment of a predetermined F-value while simultaneously controlling the target maximum temperature, thereby mitigating lipid oxidation [36]. Yu et al. observed that the processing temperature played an important role in the lipid oxidation of air-fried surimi, and an increase in temperature caused lipid oxidation [31].

The heating rate and maximum temperature of MW1 were equivalent to those of P4; however, the thermal processing level was comparatively lower in MW1 than in P4. Previous studies have demonstrated that increasing the thermal processing level at a high heating rate can enhance the fatty acid extraction coefficient. However, the total extraction coefficient of MW1 was 154.01%, which surpassed that of P4 (142.45%). Concerning the individual fatty acids, only five types of fatty acids exhibited superior extraction coefficients at P4, which substantiated the presence of non-thermal effects. Moreover, non-thermal effects can amplify the extraction coefficients of fatty acids in the flesh. The results of previous studies have also shown that microwaves improve the efficiency of lipid extraction [36,37,38]. However, their application in low-moisture target foods needs more research.

The extraction coefficients of fatty acids exhibited consistent changes following various microwave treatments, whereas the traditional heat treatment induced regular alterations in the extraction coefficients. Likewise, this could be due to the potential impact of the non-thermal effects of microwaves [39,40,41]. Hu et al. reported that non-thermal effects can significantly optimize the fatty acid composition and enhance the content of fatty acids in Atlantic salmon during pasteurization [24]. Luan et al. found that the amino acid content of sterilized rainbow trout flesh treated with microwaves was significantly higher than that under water bath treatment under nearly identical parameters [6].

## 4. Conclusions

In this study, the effects of different parameters of microwave thermal effects on the lipid quality of sterilized rainbow trout were investigated. The experimental results show that the oxidation indices and extraction coefficients of lipids exhibited regular changes under a high-temperature environment. As the heating rate increased, the lipid oxidation of flesh gradually decreased, including POVs, TBARs, and AV values. However, the lipids that could be extracted were also decreased. Higher heating rates led to less thermal damage to the cells and improved the overall quality, except the extraction coefficients of lipids.

At low heating rates, an increase in the F_0_ value caused intense oxidation as well as a reduction in measured lipid content (including total lipids and fatty acids), demonstrating that a prolonged thermal processing time destroys the lipid quality of ready-to-eat food. However, a higher F_0_ value enhanced the extraction coefficient and aggravated the oxidation partly at the high heating rate. Rapid heating not only increases the production yield but also promotes the retention of nutrients.

An elevation of the maximum temperature significantly impacted the lipid quality of the fish, including a progressive increase in oxidation indicators and a gradual reduction in extractable lipid content at a fixed F_0_. Consequently, the maximum temperature of thermal processing must be rigorously controlled in actual application.

In comparison to conventional heat treatment, microwaves can inhibit the measured indicators of lipid oxidation and preserve the lipid content, potentially due to non-thermal effects. It may be feasible to substitute the additional nutrients method, thereby further reducing product costs. Future studies are needed to investigate the influence of non-thermal effects on lipid quality.

## Figures and Tables

**Table 1:** Target parameters for thermal processing.

Processing Parameter	No	Oil Bath Temperature (°C)	Target Water Temperature (°C)	Target F_0_ (min)
Different heating rates	P_1_	128	122	3.0
	P_2_	148	122	3.0
	P_3_	168	122	3.0
	P_4_	188	122	3.0
Different thermal processing levels	P_1_	128	122	3.0
	P_5_	128	122	6.0
	P_4_	188	122	3.0
	P_6_	188	122	6.0
Different maximum water temperatures	P_4_	188	122	3.0
	P_7_	188	132	3.0
	P_6_	188	122	6.0
	P_8_	188	132	6.0
Microwave	MW1	2.50 min irradiation	No holding time	
	MW2	2.50 min irradiation	1.00 min holding time	

Note: F_0_, thermal processing level.

## Data Availability

Data are contained within the article.
